# Correction: Duan, Y.; Li, S. Effects of Plant Communities on Human Physiological Recovery and Emotional Reactions: A Comparative Onsite Survey and Photo Elicitation Study. *Int. J. Environ. Res. Public Health* 2022, *19*, 721

**DOI:** 10.3390/ijerph21121636

**Published:** 2024-12-09

**Authors:** Yifan Duan, Shuhua Li

**Affiliations:** 1 College of Landscape Architecture and Arts, Northwest A&F University, Xianyang 712100, China; dyf2010011252@163.com; 2 College of Landscape Architecture, Tsinghua University, Beijing 100084, China

## 1. Figure Legend

In the original publication [1], there was a mistake in the legend for Figure 5. There is a typo in the legend of Figure 5 (“ayer” instead of layer). The correct legend appears below. 

“Single-layer grassland”.

## 2. Table Legends

In the original publication, there were three mistakes in the legends for Tables 3–5. The original table numbers have been adjusted as a result of the addition of new table elements. The correct legends appear below.

**Table 9.** Effects of professional background and perception method.
**Table 10.** Effects of gender and plant community type on physiological and psychological indicators.
**Table 11.** Effects of professional background and plant community type on physiological and psychological indicators.

## 3. Errors in Figures

In the original publication, there were six mistakes in Figures 5–10 as published. The standard deviation (sD) and standard error (sE) were not illustrated in the original figure. All figure legends should explain what the error bars represent. Figure 5, Figure 6, Figure 7, Figure 8, Figure 9 and Figure 10 with corrections appear below.

## 4. Errors in Tables

In the original publication, there were six mistakes in Figures 5–10 as published. We have added tables. Including these statistical measures could enhance the rigor and clarity of our findings. Figures 5–10 correspond to Tables 3–8. The corrected Table 3, Table 4, Table 5, Table 6, Table 7 and Table 8 appear below.

## 5. Text Correction

There was an error in the original publication. The descriptions contained herein are not adequately precise, and the meanings they convey are susceptible to misinterpretation.

Corrections were made to the Institutional Review Board Statement and Informed Consent Statement:

**Institutional Review Board Statement:** The study was conducted in accordance with the Declaration of Helsinki, and approved by the Ethics Committee of Northwest A&F University.
**Informed Consent Statement:** The studies involving human participants were reviewed and approved by the Northwest Agriculture & Forestry University Ethics Committee. The participants provided their written informed consent to participate in this study.

The authors state that the scientific conclusions are unaffected. This correction was approved by the Academic Editor. The original publication has also been updated.

## Figures and Tables

**Figure 5:**
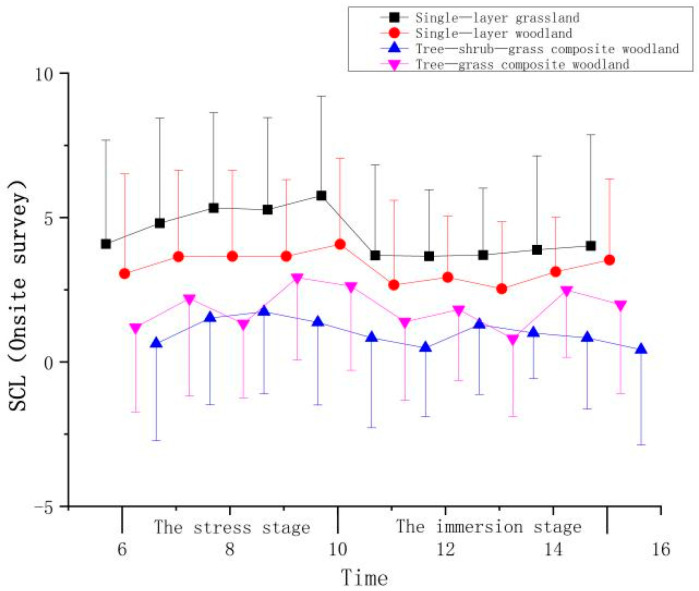
Impact of onsite surveys on participant SCL indicators.

**Figure 6:**
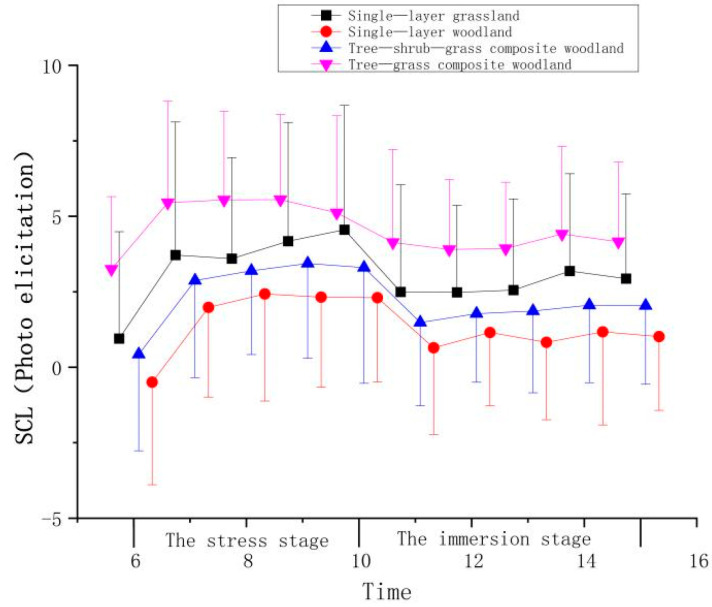
Effect of photo elicitation on the SCL indicators of the participants.

**Figure 7:**
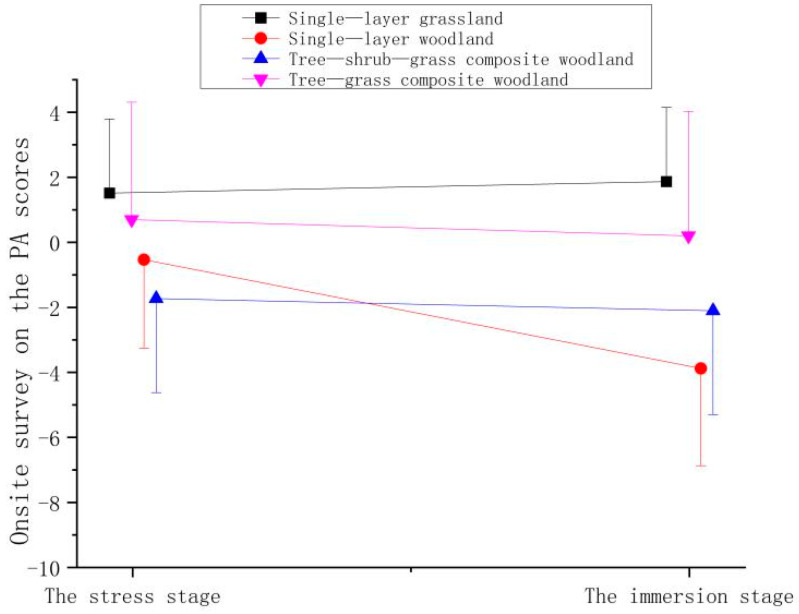
Effect of onsite surveys on participant PA scores.

**Figure 8:**
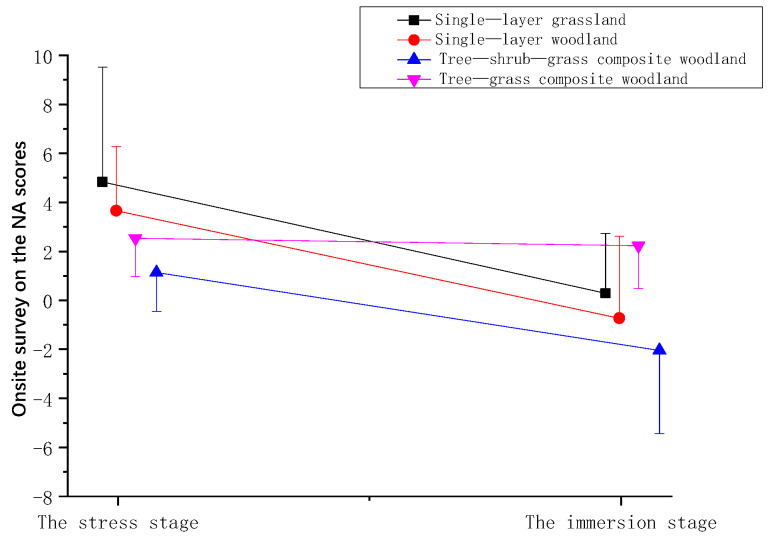
Effect of onsite surveys on the NA scores of participants.

**Figure 9:**
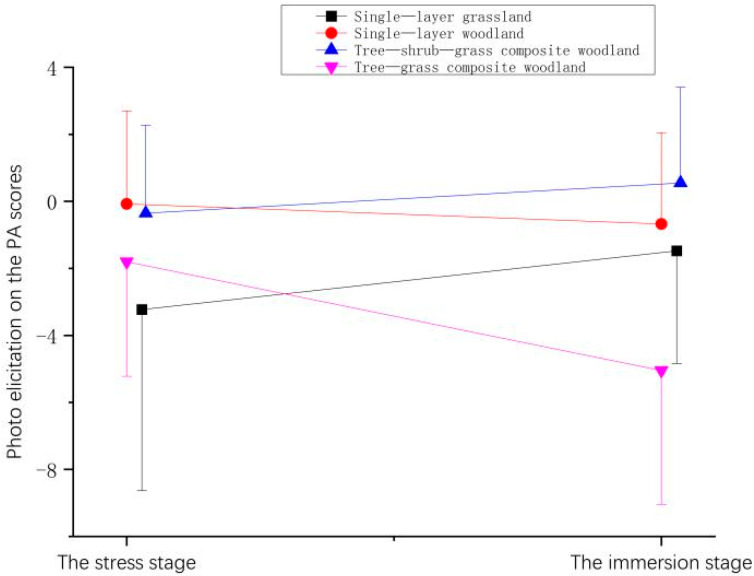
Effect of photo elicitation on the PA scores of participants.

**Figure 10:**
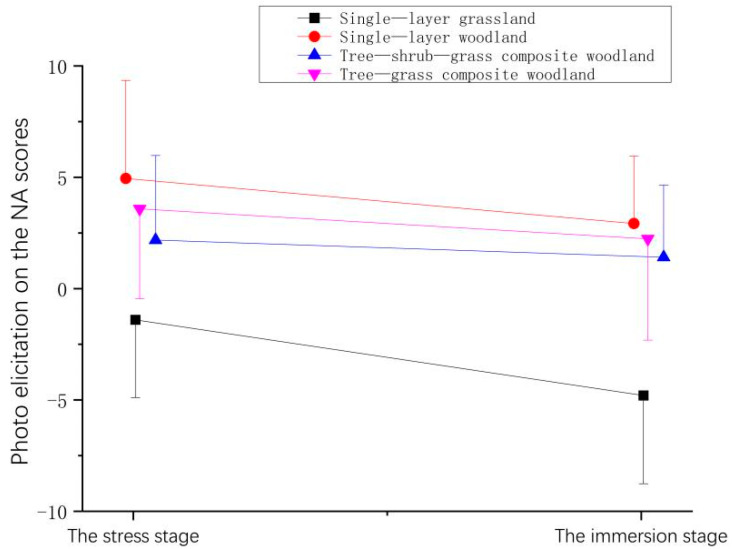
Effect of photo elicitation on the NA scores of the participants.

**Table 3:** Impact of onsite surveys on participant SCL indicators.

Stage	Time	Mean ± (SD)			
		Single-Layer Grassland	Single-Layer Woodland	Tree–Shrub–Grass Composite Woodland	Tree–Grass Composite Woodland
The stress stage	6	1.83 ± 3.59	1.29 ± 3.45	0.57 ± 3.36	0.73 ± 2.93
	7	2.55 ± 3.63	1.88 ± 3.00	1.46 ± 3.06	1.73 ± 3.39
	8	3.08 ± 3.30	1.89 ± 2.97	1.68 ± 2.85	0.86 ± 2.57
	9	3.02 ± 3.18	1.89 ± 2.65	1.31 ± 2.86	2.45 ± 2.86
	10	3.51 ± 3.44	2.30 ± 2.97	0.77 ± 3.11	2.16 ± 2.92
The immersion stage	11	1.44 ± 3.13	0.90 ± 2.93	0.42 ± 2.39	0.92 ± 2.73
	12	1.41 ± 2.29	1.16 ± 2.12	1.23 ± 2.42	1.35 ± 2.46
	13	1.45 ± 2.31	0.77 ± 2.32	0.94 ± 1.58	0.33 ± 2.69
	14	1.63 ± 3.25	1.36 ± 1.88	0.77 ± 2.47	2.02 ± 2.34
	15	1.77 ± 3.84	1.76 ± 2.81	0.36 ± 3.30	1.52 ± 3.08

**Table 4:** Effect of photo elicitation on the SCL indicators of the participants.

Stage	Time	Mean ± (SD)			
		Single-Layer Grassland	Single-Layer Woodland	Tree–Shrub–Grass Composite Woodland	Tree–Grass Composite Woodland
The stress stage	6	0.20 ± 3.54	−0.11 ± 3.40	0.01 ± 3.19	0.48 ± 2.40
	7	2.97 ± 4.40	2.37 ± 2.97	2.46 ± 3.22	2.68 ± 3.36
	8	2.85 ± 3.35	2.81 ± 3.55	2.78 ± 2.78	2.78 ± 2.93
	9	3.43 ± 3.93	2.71 ± 2.99	3.03 ± 3.14	2.78 ± 2.83
	10	3.81 ± 4.12	2.69 ± 2.80	2.89 ± 3.84	2.35 ± 3.21
The immersion stage	11	1.75 ± 3.55	1.03 ± 2.87	1.07 ± 2.77	1.37 ± 3.07
	12	1.73 ± 2.88	1.54 ± 2.42	1.36 ± 2.28	1.13 ± 2.31
	13	1.81 ± 3.02	1.21 ± 2.57	1.45 ± 2.72	1.17 ± 2.19
	14	2.44 ± 3.22	1.56 ± 3.08	1.65 ± 2.57	1.65 ± 2.90
	15	2.19 ± 2.81	1.40 ± 2.44	1.63 ± 2.61	1.39 ± 2.64

**Table 5:** Effect of onsite surveys on participant PA scores.

Stage	Mean ± (SD)			
	Single-Layer Grassland	Single-Layer Woodland	Tree–Shrub–Grass Composite Woodland	Tree–Grass Composite Woodland
The stress stage	−0.18 ± 2.27	−0.50 ± 2.72	−0.80 ± 2.90	−1.18 ± 3.61
The immersion stage	0.18 ± 2.28	−3.85 ± 2.99	−1.18 ± 3.20	−1.68 ± 3.83

**Table 6:** Effect of onsite surveys on the NA scores of participants.

Stage	Mean ± (SD)			
	Single-Layer Grassland	Single-Layer Woodland	Tree–Shrub–Grass Composite Woodland	Tree–Grass Composite Woodland
The stress stage	3.13 ± 4.68	1.83 ± 2.62	0.28 ± 1.58	0.88 ± 1.56
The immersion stage	−1.4 ± 2.45	−2.55 ± 3.34	−2.90 ± 3.40	0.58 ± 1.75

**Table 7:** Effect of photo elicitation on the PA scores of participants.

Stage	Mean ± (SD)			
	Single-Layer Grassland	Single-Layer Woodland	Tree–Shrub–Grass Composite Woodland	Tree–Grass Composite Woodland
The stress stage	−3.25 ± 5.41	−0.08 ± 2.77	−0.38 ± 2.62	−1.80 ± 3.42
The immersion stage	−1.50 ± 3.36	−0.68 ± 2.72	0.53 ± 2.85	−5.05 ± 3.99

**Table 8:** Effect of photo elicitation on the NA scores of the participants.

Stage	Mean ± (SD)			
	Single-Layer Grassland	Single-Layer Woodland	Tree–Shrub–Grass Composite Woodland	Tree–Grass Composite Woodland
The stress stage	−1.35 ± 3.50	2.65 ± 4.40	1.65 ± 3.79	1.58 ± 4.04
The immersion stage	−4.75 ± 3.97	0.63 ± 3.03	0.88 ± 3.24	0.23 ± 4.55
